# Testing the Two-Step Model of Plant Root Microbiome Acquisition Under Multiple Plant Species and Soil Sources

**DOI:** 10.3389/fmicb.2020.542742

**Published:** 2020-10-09

**Authors:** Hugo R. Barajas, Shamayim Martínez-Sánchez, Miguel F. Romero, Cristóbal Hernández Álvarez, Luis Servín-González, Mariana Peimbert, Rocío Cruz-Ortega, Felipe García-Oliva, Luis D. Alcaraz

**Affiliations:** ^1^Departamento de Biología Celular, Facultad de Ciencias, Universidad Nacional Autónoma de México, Mexico City, Mexico; ^2^Departamento de Biología Molecular y Biotecnología, Instituto de Investigaciones Biomédicas, Universidad Nacional Autónoma de México, Mexico City, Mexico; ^3^Departamento de Ciencias Naturales, Unidad Cuajimalpa, Universidad Autónoma Metropolitana, Mexico City, Mexico; ^4^Laboratorio de Alelopatía, Departamento de Ecología Funcional, Instituto de Ecología, Universidad Nacional Autónoma de México, Mexico City, Mexico; ^5^Instituto de Investigaciones en Ecosistemas y Sustentabilidad, Universidad Nacional Autónoma de México, Morelia, Mexico

**Keywords:** plant microbiome, soil microbiome, rhizosphere metagenomics, core metagenome, domesticated plants, ruderal plants, common garden experiment

## Abstract

The two-step model for plant root microbiomes considers soil as the primary microbial source. Active selection of the plant’s bacterial inhabitants results in a biodiversity decrease toward roots. We collected sixteen samples of *in situ* ruderal plant roots and their soils and used these soils as the main microbial input for single genotype tomatoes grown in a greenhouse. Our main goal was to test the soil influence in the structuring of rhizosphere microbiomes, minimizing environmental variability, while testing multiple plant species. We massively sequenced the 16S rRNA and shotgun metagenomes of the soils, *in situ* plants, and tomato roots. We identified a total of 271,940 bacterial operational taxonomic units (OTUs) within the soils, rhizosphere and endospheric microbiomes. We annotated by homology a total of 411,432 (13.07%) of the metagenome predicted proteins. Tomato roots did follow the two-step model with lower α-diversity than soil, while ruderal plants did not. Surprisingly, ruderal plants are probably working as a microenvironmental oasis providing moisture and plant-derived nutrients, supporting larger α-diversity. Ruderal plants and their soils are closer according to their microbiome community composition than tomato and its soil, based on OTUs and protein comparisons. We expected that tomato β-diversity clustered together with their soil, if it is the main rhizosphere microbiome structuring factor. However, tomato microbiome β-diversity was associated with plant genotype in most samples (81.2%), also supported by a larger set of enriched proteins in tomato rhizosphere than soil or ruderals. The most abundant bacteria found in soils was the Actinobacteria *Solirubrobacter soli*, ruderals were dominated by the Proteobacteria *Sphingomonas* sp. URGHD0057, and tomato mainly by the Bacteroidetes *Ohtaekwangia koreensis*, *Flavobacterium terrae, Niastella vici*, and *Chryseolinea serpens.* We calculated a metagenomic tomato root core of 51 bacterial genera and 2,762 proteins, which could be the basis for microbiome-oriented plant breeding programs. We attributed a larger diversity in ruderal plants roots exudates as an effect of the moisture and nutrient acting as a microbial harbor. The tomato and ruderal metagenomic differences are probably due to plant domestication trade-offs, impacting plant-bacteria interactions.

## Introduction

Soil and plant root-associated bacteria are relevant for plant health, which has already been noticed in the beginning of the 20th century (Hiltner, 1904). It has been hypothesized that the microbiome could be related to crop quality (Hartmann et al., 2008). Soil is the most diverse microbial ecosystem, with up to 10^11^ bacterial cells per gram (Roesch et al., 2007). Soil properties such as pH, nutrient content, or moisture, and plant species can drive the soil microbiome composition (Fierer and Jackson, 2006; Lauber et al., 2009; Schlaeppi et al., 2014). Plants and soil interact at the rhizosphere, defined as the millimetric soil layer attached to plant roots. Plants play an active role in selecting their microbial inhabitants through root exudates, accounting from 5 to 20% of the photosynthetically fixed carbon and used by the microbes (Marschner et al., 2004). Plant-microbe interactions mainly occur in the rhizosphere (Berendsen et al., 2012). Some other known factors affecting the root microbial community structure are plant developmental stage (Inceoğlu et al., 2011), pathogen presence (Tian et al., 2015), and soil characteristics (Lundberg et al., 2012; Edwards et al., 2015). Plant-microbiome interaction has documented effects on plant growth and health; for example, the root microbiome composition has been associated with biomass increase in *Arabidopsis thaliana* (Swenson et al., 2000) and can also affect flowering time (Panke-Buisse et al., 2015).

A study of the *A. thaliana* microbiome using hundreds of plants and two soil sources concluded that root bacterial communities were strongly influenced by soil type (Lundberg et al., 2012). Microbial diversity was reduced in the rhizosphere compared to the surrounding soil, suggesting that plants filter and recruit a microbiome subset; these observations have led to the two-step model of microbiome selection (Bulgarelli et al., 2012, 2013). This model considers soil abiotic properties in the soil microbiome (first step), and specific plant-derived rhizodeposits contribute to selecting differential microbes in the rhizosphere and the endosphere (second step) (Bulgarelli et al., 2013). In the two-step model, α-diversity decreased in the following order: soils > rhizosphere > endosphere (Bulgarelli et al., 2013). However, a global-scale meta-analysis has reported that root microbiomes of multiple plant species (domesticated and wild) have a more substantial diversity than soils (Thompson et al., 2017).

**FIGURE 1:**
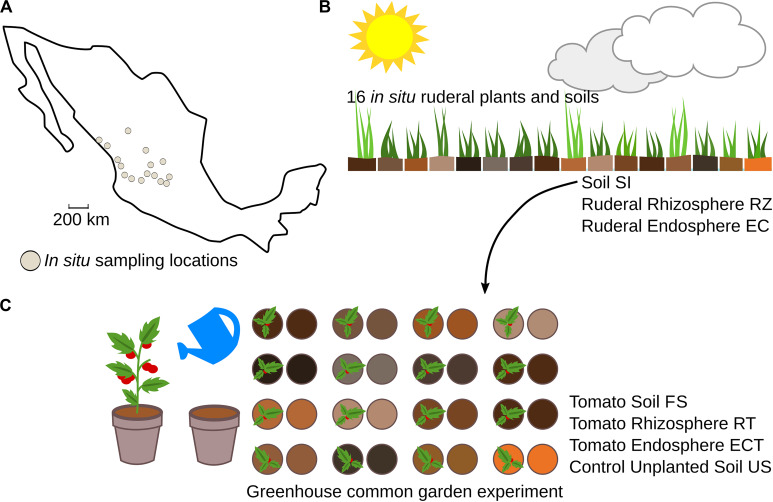
Experimental overview of the work. **(A)** *In situ* sampling locations; sampling points were selected according to edaphological charts. **(B)** The *in situ* plants were dependent on the weather and local environmental conditions, and we collected soil samples (SI) and roots of the dominant plant species in each locality. We extracted the rhizosphere (RZ) and endosphere (EC) metagenomic DNA. **(C)** A common garden experiment was conducted in a greenhouse; the soil (SI) was used as a microbial inoculum to reduce environmental variability. Plant diversity was eliminated using tomato, with constant watering, and finally, we collected roots (RT), endosphere (ECT), final soil (FS), and control unplanted soil (US).

This work explores the bacterial diversity by 16S rRNA gene massive amplicon sequencing and whole shotgun metagenomes to predict the protein diversity of 16 geochemically distinct Mexican soils, collected over a large geographical scale (Figure 1A and Table 1). The collected soils were chosen based on country-wide edaphological charts (INEGI, 2014). We explored the role of soil in microbiome structuring of *in situ* ruderal plants, growing above the collected soils with multiple species and at several plant developmental stages. The collected soils were used as the substrate in a greenhouse experiment for growing tomatoes (*Solanum lycopersicum*), eliminating plant genotype variability as well as developmental, climatic, and watering variables. Finally, testing diverse soil groups allowed us to explore the tomato core root microbiome, which follows the two-step model for root microbiome selection. The ruderal plants do not follow the two-step model and have a larger diversity than their source soils.

**TABLE 1:** Soil edaphological classification, abiotic properties, and tomato biomass production.

**Site**	**Soil group**	**pH (SI)**	**pH (SF)**	**Total N (mg/g) (SI)**	**Total N (mg/g) (SF)**	**Total C (mg/g) (SI)**	**Total C (mg/g) (SF)**	**Total P (mg/g) (SI)**	**Total P (mg/g) (SF)**	**Tomato biomass (SF) (dry weight in g)**	**AI**	**Altitude**	**Latitude**	**Longitude**	**Collection Date**
JAL4	Phaozem	7.2	6.0	0.623	11.721	16.503	31.003	0.009	0.16	0.221	48.8	1490	20.849	−103.855	11/8/2014
NAY2	Cambisol	5.7	6.1	6.83	16.667	19.347	22.086	0.453	0.284	0.083	56.5	38	21.836	−105.083	11/8/2014
JAL3	Luvisol	6.5	6.8	3.99	17.799	32.863	25.683	0.475	0.263	0.090	46.1	1862	20.821	−102.798	11/6/2014
NAY3	Cambisol	6.9	6.9	2.027	11.584	9.924	13.569	0.157	0.197	0.204	47.1	4	22.142	−105.261	11/8/2014
SLP1	Kastanozem	6.9	7.8	9.702	5.898	79.348	146.37	0.447	0.287	0.767	22.1	2130	21.988	−101.277	11/10/2014
ZAC1	Cambisol	5.0	7.2	6.433	12.961	4.921	5.117	0.324	0.347	0.017	25	2221	22.818	−102.696	11/10/2014
SIN1	Phaozem	7.3	7.4	7.523	2.095	16.862	24.452	0.377	0.42	0.223	29.8	23	22.988	−105.880	11/8/2014
JAL5	Lithosol	7.4	7.9	2.158	24.011	5.067	5.427	0.235	0.192	0.199	44	1328	21.179	−104.540	11/8/2014
JAL1	Planosol	7.5	7.6	1.892	1.339	9.482	14.425	0.118	0.164	0.313	32.9	1810	21.162	−101.853	11/6/2014
AGS1	Planosol	8.0	8.3	5.671	12.46	19.616	22.01	0.268	0.28	0.269	29.2	2059	21.828	−102.121	11/10/2014
GTO1	Vertisol	8.3	8.6	12.298	13.387	22.733	18.546	0.378	0.323	0.259	32.9	1708	20.580	−100.947	11/11/2014
GTO3	Vertisol	8.4	8.4	2.624	6.008	10.559	10.378	0.271	0.323	0.180	29.2	2012	20.897	−100.674	11/11/2014
JAL2	Phaozem	8.6	9.0	2.075	8.646	14.929	13.285	0.01	0.336	0.219	38.2	1791	21.253	−102.298	11/6/2014
GTO2	Vertisol	8.6	8.7	7.089	13.948	18.038	17.722	0.4	0.417	0.156	33.8	1710	20.721	−101.329	11/11/2014
DGO1	Planosol	8.9	8.9	5.018	10.838	23.36	23.773	0.251	0.248	0.156	33.8	1906	24.006	−104.385	9/11/2014
SIN2	Regosol	9.1	8.8	18.578	4.91	49.131	50.98	1.103	0.157	0.314	29	10	23.293	−106.479	9/11/2014

*Soils were classified according to the United Nations’ FAO edaphological charts. The main attributes of the soils used in this work are sampling location, altitude (m.a.s.l.), Lang’s Aridity Index (AI), and contents of carbon, nitrogen, and phosphorus. Initial refers to source soil (SI) and final to common garden experiment output soil (FS) measurements.*

## Results

### Soil Geochemical Description

Total nutrient concentration (C, N, P), pH, and Lang’s aridity index were calculated and considered as soil abiotic properties (Table 1). With the common garden experiment, we increased soil biological activity, reflected in the N and C overall increases after the experiment. We observed an increase in N concentrations in 12/16 samples, while total C increased in 11/16 samples, and P decreased in 7/16 samples (Table 1). Another explanation for the soil carbon enrichment is by plant root exudates (Canarini et al., 2019). Tomatoes planted in SLP1 and SIN2 exhibited a reduction in their total N concentrations; in SLP1, this is explained as plant biomass generation, and in SIN2, a coastal dune N was probably drained through watering (Table 1 and Supplementary Figure S2). Only two samples changed their pH profiles (Table 1). Ordination analysis showed clustering apart of source soils (SI) from final greenhouse soils (FS) and evidenced the modifications derived from the common garden experiment (Supplementary Figure S2A and Table 1).

### Microbiome Diversity in the Source Soils, Ruderal Plants, and Tomatoes

A total of 106 amplicon libraries (16S rRNA gene V3-V4) were sequenced (Figure 1 and Supplementary Table S1). After quality control and assembly, 5,211,969 sequences were recovered. Subsequently 2,570,541 operational taxonomic units (OTUs; 97% identity) were clustered. After discarding singleton, mitochondrial, chloroplast, chimeras, and non-matching sequences, a total of 271,940 OTUs were the base for further analysis. The average *in situ* source soil (SI) OTU number (SI = 2,143) was lower than that in ruderal plants rhizospheres (RZ) and endosphere (EC) (RZ = 18,158; EC = 19,885 OTUs). Common garden final soils (FS) and control unplanted soils (US) had similar OTU averages (FS = 3,084; US = 2,882). The control soils (US) were pots with unplanted soil and output of the greenhouse experiment, thus receiving the same watering and homogeneous environmental conditions as final soils (FS), and tomato root microbiomes (RT and ECT). The rationale behind the common garden experiment is to impose a homogeneous treatment for all experimental units, in this case, regular watering and the same temperature, thereby eliminating local climatic variables of soil (temperature, humidity, altitude, precipitation). Lang’s aridity index is based on the historical data of precipitation and temperature in a site. The aridity index of soils is disrupted in the common garden experiment where the environmental conditions are homogenized. We hypothesize that control soils (US) have a larger number of OTUs than source soils (SI) either by reactivation of biological activity, increasing the abundance of microorganisms, and the proliferation of external sources of microbes such as the ones carried through air and water. Tomato rhizosphere (RT) and endosphere (ECT) samples had a higher OTU average than the SI, but a smaller average compared to FS (RT = 2,474 OTUs, EC = 2,088) (Supplementary Table S2).

**FIGURE 2:**
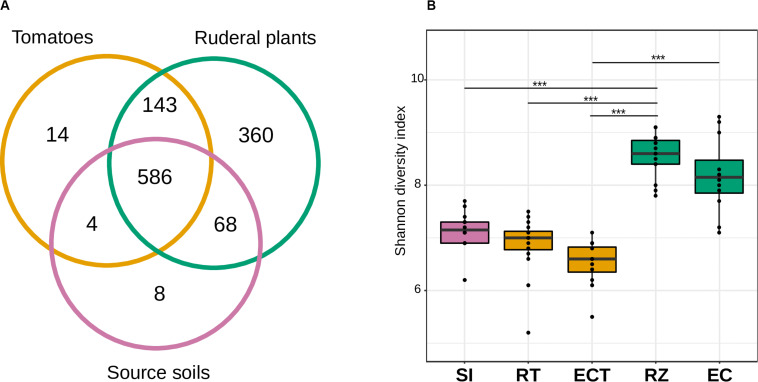
Alpha diversity and richness of the soil, rhizosphere, and endosphere of tomato and ruderal plants. **(A)** Venn diagram showing the number of shared bacterial genera in roots (endosphere + rhizosphere) and soils. **(B)** Boxplots showing the OTU Shannon diversity index (H&2032;) of source soils (SI), tomato rhizosphere (RT), tomato endosphere (ECT), *in situ* plants rhizosphere (RZ), and *in situ* ruderal plant endosphere (EC).

We found 586 shared bacterial genera between soils and roots (rhizosphere and endosphere) of tomatoes and ruderal plants (Figure 2A). The source soils had eight unique genera and shared most (98.78%) of their microbes with tomatoes or ruderal plants. The largest amount of unique genera (46.21%) was found for the ruderal plants, sharing the most bacterial genera with the tomato and the soils (53.78%). The tomato root microbiomes had 14 unique bacterial genera (1.9%), four were only shared with soils (0.53%), while most genera were shared with soils and ruderal plants (97.53%). The unique bacterial members found in plants and not found in soil may be a product of vertical inheritance of the plant microbiome (e.g., seed endophytes, Truyens et al., 2015; Shade et al., 2017). Another of our analyses showed that the tomato core microbiome had 51 bacterial genera, while ruderal plants and core soils had 187 and 16 bacterial genera, respectively. Cores were defined as detected genera in all of the sample types compared (RT, soils, and RZ). Complete information on unique and shared OTUs is available (Supplementary Table S3). In comparison to our result, another study identified a bacterial tomato rhizosphere core microbiome composed of 68 orders using different tomato cultivars in a single soil, and 27 orders using a single tomato genotype in five soils (Cheng et al., 2020).

### Ruderal Plants Rhizospheres Harbored a Larger Bacterial Diversity Than Soils and Tomatoes

We analyzed the α-diversity of soils, rhizospheres, and endosphere microbiomes through the Shannon diversity index (H′). Multiple studies have confirmed the two-step model of root microbiomes (Lundberg et al., 2012; Bulgarelli et al., 2013; Edwards et al., 2015). Here, we found that soils (H′ = 6.1–7.6) were more diverse than the tomato rhizosphere (H′ = 5.2–7.4) or the tomato endosphere (H′ = 5.5–7.1), thus fitting the two-step model for microbiome selection. However, when comparing the soil to the ruderal plant root microbiomes, higher H′ values were observed in the rhizosphere (H′ = 7.4–9.1) and even in their endosphere microbiomes (H′ = 7.0–9.2) compared to their soils (H′ = 6.1–7.6), not adjusting to the two-step model (Figure 2B and Supplementary Figure S3, Supplementary Table S2).

Ruderal plants might have a larger α-diversity because of the rhizosphere micro-environmental conditions, analogs to an oasis in the dry soil. However, previous reports show a larger diversity in rhizospheres than in soils comparing different biomes (Thompson et al., 2017). Marasco et al. (2018) found a higher 16S rRNA copies (qPCR) in the rhizosphere and rhizosheath than soil, of three species of dune colonizing speargrasses (*Stipagrostis sabulicola, S. seelyae, and Cladoraphis spinosa*). The larger rhizosphere and rhizoheath suggest an increase in microbial abundance and activity in these plants influenced microniches compared to soils. They also found that *S. seelyae* possessed a more substantial species richness in the rhizosphere than the bulk soil. Another study found that plants of *Caragana microphylla* can host rhizospheric microbial communities with larger Shannon diversity values in comparison to their bulk soils in particular sites or depending on the type of dunes (Gao et al., 2019). Additionally, some considerations must be made for ruderal plants since they are present in environments where they are not the only plant species but part of a plant community that could be broadening the rhizosphere effect. Different plant species or genotypes, as well as plant age, have been reported to attract specific bacterial communities (Baudoin et al., 2002; Marschner et al., 2004; Micallef et al., 2009). Additionally, plant communities and their richness and diversity growing in the soil affects belowground microbial community diversity, biomass, and respiration rates, thereby impacting plant diversity (Wu et al., 2019). Current agricultural management includes practices such as fertilizer-driven production, which decreases the importance of plant-microbe interactions when scavenging for nutrients (van der Heijden et al., 2008). The larger microbial diversity observed in ruderal plants shows the commitment of wild plants to their microbes, fostering plant-microbe relationships which are not observed in domesticated cultivars (Wissuwa et al., 2009). We have previously tested other non-domesticated plants, such as the aquatic carnivorous bladderwort *Utricularia gibba* (Alcaraz et al., 2016) and the bryophyte species *Marchantia polymorpha* and *M. paleacea* (Alcaraz et al., 2018); both showed less diversity in their root analogs (bladders, and rhizoids) than their soil sources, supporting the two-step model. The *Marchantia* microbiomes even allowed us to perform an extreme microbial selection due to the *in vitro* propagation of these plants, highlighting a reduced core of closely related microbial inhabitants (Alcaraz et al., 2018). Testing multiple plants, wild and domesticated, could reduce the gaps in understanding the microbiome structure loss as a domestication trade-off.

### Plant Driven Selection of Bacterial Root Colonizers in Tomatoes and Ruderal Plants

**FIGURE 3:**
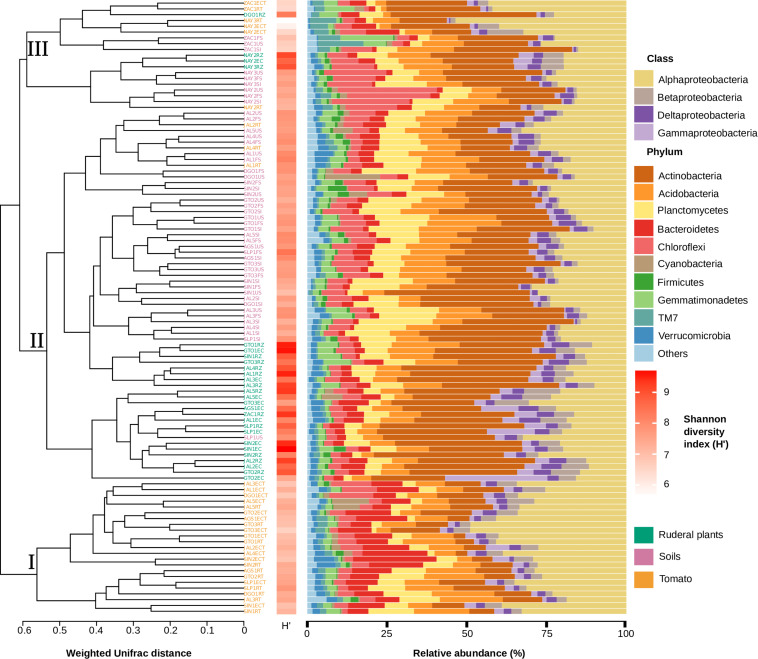
Host genotype and soil influence on microbial community structure (16S rRNA gene). On the left, a weighted Unifrac dendrogram shows α-diversity and phylogenetic similarity between soil, tomato, and ruderal plants. Each location is indicated at the dendrogram terminal nodes with a three-letter key for sampling location and suffix indicating type: initial source soil (SI), final soil (FS), unplanted soil (US), tomato rhizosphere (RT), tomato endosphere (ECT), ruderal plant rhizosphere (RZ), and ruderal plant endosphere (EC). Phyla diversity (H&2032;) in each sampled microbiome is shown as a horizontal heatmap. Bar plots show bacterial phyla relative abundance in each sample. Proteobacteria are shown at the class level in the bar plots.

We performed a α-diversity analysis based on the weighted UniFrac community distance matrix to dissect the role of soil in the establishment and structure of rhizo and endosphere microbiomes in both ruderal and *S. lycopersicum* plants (Figure 3). We observed that the microbiome (16S rRNA gene) distribution was largely driven by the host species. The weighted UniFrac dendrogram grouped the samples into three major clusters: Cluster (I) contains only tomato-associated microbiomes, cluster (II) includes soil and ruderal plant microbiomes, and a mixed cluster (III) includes soil, tomato, and ruderal plant microbiomes (Figure 3). The clustering of the three groups is supported by ANOSIM (*R* = 0.7257; *p* < 0.001; 999 permutations). Pairwise distance values were calculated between every sample in the weighted UniFrac dendrogram to evaluate the distance patterns and cohesion found inside and between each of the described clusters. The average internal distances were 0.5041 for cluster (I), 0.5058 for cluster (II), and 0.4787 for the cluster (III). The measured distance between any terminal node of cluster I against any tip in either cluster (II) or (III) was 0.6608. Most of the tomato samples were closer to each other than to their source soils. The tomato-associated cluster (Figure 3, cluster I) grouped 10/16 tomato rhizospheres, along with 13/16 of the tomato endospheres, suggesting a plant genotype-dependent role in root microbiome establishment. The closer α-diversity distance of ruderal plants to their soils, compared to tomatoes (Figure 3), showed the tomato host genotype associated microbiome selection having a larger effect than soil, lowering its overall α-diversity in a probable outcome of domestication trade-offs. A comparison of maize, its ancestor teosinte, and other Poaceae rhizosphere microbiomes showed correlations between microbiomes and host evolutionary distances (Bouffaud et al., 2014). The few tomatoes and ruderal samples that clustered closer to their source soils, were remarkably acid soils, indicating pH properties as microbiome structuring factor, as shown before (Fierer and Jackson, 2006; Männistö et al., 2007; Table 1).

### Microbiome Phylogenetic Assignments and Differential Taxa in Soil, Ruderals, and Tomato

*In situ* samples of soils and ruderal plants were dominated by Actinobacteria, with a significantly (ANOVA *p* < 2e-16) lower abundance in tomato roots (Figure 3, Supplementary Figure S4 and Supplementary Table S5). Using the tomato (fixed plant genotype), we imposed a selective factor, since the plant-derived chemotactic signals and photosynthates should be similar, independent of the soil. Proteobacteria were significantly enriched in tomatoes (ANOVA *p* < 1.82e-15) compared to soils and ruderal plants. It seems that plants such as tomatoes as other agricultural species favor Proteobacteria (Correa-Galeote et al., 2018; Cordero et al., 2020), while ruderals and soils depend upon Actinobacteria. The class α-Proteobacteria was the most abundant in tomatoes, with significant enrichment (ANOVA *p* < 2e-16) compared to ruderal plants and soils. The β, γ, and δ-Proteobacteria were more abundant in ruderal plants (*p* < 0.05) than in tomatoes and soils. Bacteroidetes were enriched in tomato roots (ANOVA *p* < 1.34e-15) when compared to soils and ruderal plants (Figure 3, Supplementary Figure S4, and Supplementary Table S5). Each plant can attract and select specific microorganisms depending on plant-genotype-dependent chemical formulation of rhizodeposits and cell wall features, resulting in specificity for microbiome selection (Schlaeppi et al., 2014; Bulgarelli et al., 2015; Fitzpatrick et al., 2018). Additionally, *in situ* natural variations in the climatic conditions were reduced in the common garden experiment, tomato plants watered regularly, and minimized climatic variation. The larger abundance of Actinobacteria has practical explanations in plant interactions; there are reports of its use as biocontrol agents isolated from soil and rhizospheres, and they are secondary metabolite producers such as antibiotics or plant growth-promoting molecules such as indole acetic acid (El-Tarabily et al., 2009; Brader et al., 2014; Sreevidya et al., 2016). Actinobacteria differential abundance in both soils and ruderal plants can also be a product of environmental water limitations. Aridity increases the proportions of Actinobacteria in arid soils, while humid sites usually have larger Proteobacteria abundances (Neilson et al., 2017), probably because Proteobacteria have faster duplication times than Actinobacteria (Ramin and Allison, 2019).

We used DESeq2 to compare and identify significantly (*p* < 0.01, Bonferroni corrected) enriched OTUs (Supplementary Table S6). The overall diversity decrease in the tomato roots is consistent with the enrichment of certain bacterial groups capable of close plant interactions through specific molecular mechanisms (e.g., chemotaxis responsive, plant degradation enzymes) (Bais et al., 2006; Compant et al., 2010). We found six differential OTUs assigned as *Sphingobium, Caulobacter, Asticcacaulis, Arthrospira*, and *Kaistobacter* in the tomato rhizospheres compared to their source soils (Supplementary Figure S5A). These bacterial genera have been isolated from sources such as freshwater (Chen et al., 2013), soil (Costa et al., 2006), and rhizospheres (Young et al., 2008; Schreiter et al., 2014; Yang et al., 2016). The genera *Caulobacter* and *Asticcacaulis* are characterized by having at least one appendage or prostheca that protrudes from the cell envelope and can play a role in adhesion to solid substrates (Poindexter, 1981; Ong et al., 1990), a helpful attribute for the colonization of plant roots (Wheatley and Poole, 2018). Additionally, *Caulobacter* has been described as a hub taxa in the phyllosphere of *Arabidopsis thaliana* (Agler et al., 2016), remarking the possible importance of these taxa in plant-associated microbial communities. In contrast, in the endospheres, we found 14 enriched OTUs belonging to the same genera present in the rhizosphere and *Agrobacterium* and *Lacibacter* (Supplementary Figure S5B). The presence of OTUs assigned to the families Sphingomonadaceae and Bradyrhizobiaceae in roots of *S. lycopersicum* has been reported previously. Both Sphingomonadaceae and Bradyrhizobiaceae OTUs were reduced with plants inoculated with the pathogen *Phytophthora parasitica*, compared to healthy plants (Larousse et al., 2017). We found an overrepresentation of some *Sphingobium* and *Rhizobium* species, suggesting that their abundance could be used as a plant health proxy since we did not observe root rot symptoms in any of our individuals, as in other studies with healthy tomato plants (Satour and Butler, 1967; Lee et al., 2019). Moreover, in a previous work describing tomato roots, microbiomes, *Sphingomonas*, and *Sphingobium* were detected in more than 50% of the 16S rRNA gene OTUs (Lee et al., 2016). *Sphingobium* has been observed as the dominant genus in tomato roots elsewhere (Pii et al., 2016). The comparison between ruderal plant roots and soils showed 45 differentially abundant OTUs in the rhizospheres (Supplementary Figure S5C) and 31 enriched OTUs in the endosphere (Supplementary Figure S5D), most belonging to Actinobacteria (Supplementary Table S6). Additionally, we compared the sets of soils and their controls (Figure 1) and did not find any shared OTUs whose abundance differed significantly. Most of the ruderals in our study were grasses (Poaceae; Supplementary Figure S1), and recently it was reported that grasses rhizospheres were enriched in Actinobacteria under drought conditions (Naylor et al., 2017). Also, the loss of Actinobacteria abundance in tomato, a domesticated crop, compared to the soils and ruderal plants suggests that it could be a domestication trade-off, as previously suggested by a correlation between microbiome structure and host evolutionary history (Redford et al., 2010; Peiffer et al., 2013; Bouffaud et al., 2014).

### Shotgun Metagenomic Diversity in Source Soils, Ruderal Plant Rhizospheres, and Tomato Rhizospheres

We sequenced 50.1 Gb in a total of 17 SI, RT, and RZ metagenomes. After quality control, we obtained 464,372,598 high-quality paired-end reads (μ = 27,316,035 ± 2,943,233 per sample), which were used as input to an assembly that yielded 12,677,118 contigs (μ = 745,713 ± 366,001 per sample), with an average N50 of 176 ± 51 bp and the longest contig average length of 45,645 bp. Subsequently, we were able to compute a total of 12,272,971 predicted peptides (μ = 708,835 ± 332,770 per sample) (Supplementary Table S7). Protein redundancy was reduced using proxy-genes of matches to known proteins and protein-clustering alignments (70% identity). After clustering and matching, protein annotation was performed using the M5NR database (see “Materials and Methods”), resulting in 3,147,929 proteins; only 411,432 (13.07%) were annotated based on homology against the M5NR database.

**FIGURE 4:**
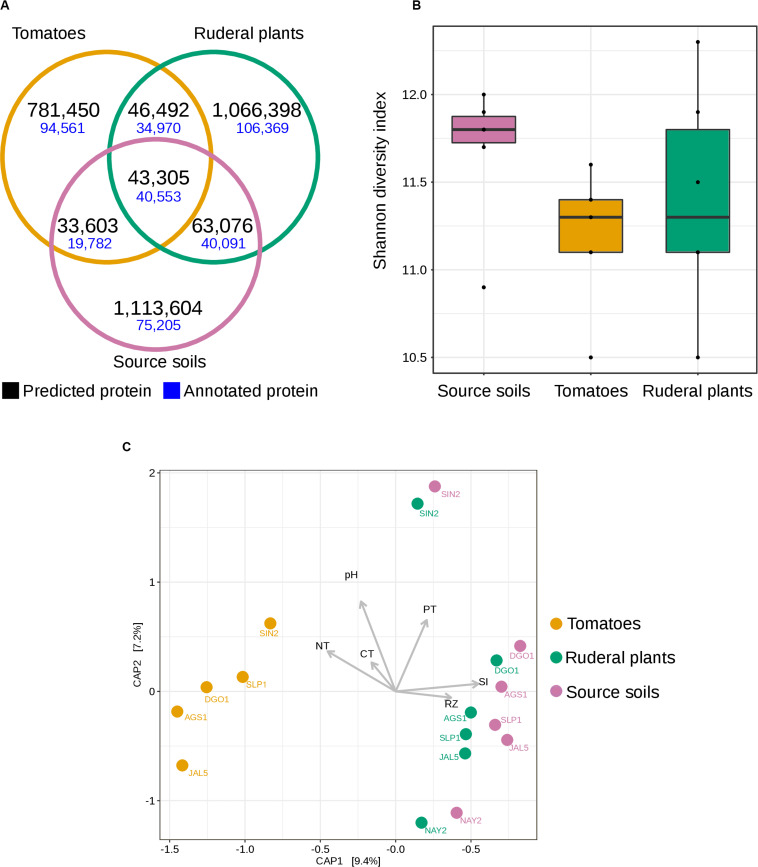
Shotgun metagenomics diversity of soil and rhizosphere microbial communities. **(A)** Venn diagram showing the number of shared and unique annotated protein families (70% identity) in soil and rhizosphere. **(B)** Boxplots showing the Shannon diversity index based on the total number of predicted proteins in soil and rhizosphere. **(C)** Constrained analysis of principal coordinates (CAP), calculated from the total number of predicted proteins for all sequenced soil and rhizosphere metagenomes using Bray-Curtis dissimilarity. Vectors display the environmental factors: CT, Total Carbon concentration; NT, Total Nitrogen concentration; PT, Total Phosphorus concentration; RZ, ruderal plant rhizosphere; SI, Initial soil.

We compared the shared set of proteins between soils, ruderals, and tomatoes, resulting in a set of 43,305 proteins detected at least once for every sample type (Figure 4A). Most of the union set proteins (93%) were annotated. Tomatoes shared with the soils 8.49% of their predicted proteins, while ruderal plants shared 8.72% of the identified peptides with the soil. Tomatoes shared more coding genes with ruderal plants (8.85%) than with soil (8.49%) (Figure 4A). Different sets of proteins for each sample showed the largest novelty in soil (88.83%), followed by ruderal plants (87.46%) and tomatoes (86.36%) (Figure 4A). Although the largest number of unique proteins could be the result of an enthusiastic computer prediction, it was interesting that the tomato had the largest amount of annotated proteins (12.10%) compared to ruderals (9.97%) and soils (6.75%), maybe reflecting the larger previous genomic information in agricultural microbes, being scarcer in wild plants, and the soil microbes (Figure 4A). Complete lists of identified proteins are available as Supplementary Material (Supplementary Table S8).

We compared the protein α-diversity using the Shannon diversity index (H′) based on the total number of predicted proteins (Supplementary Table S9). Soil diversity had a higher median (H′ = 11.8) than tomato diversity (H′ = 11.3) and ruderal plant diversity (H′ = 11.3), without significant differences (Figure 4B and Supplementary Figure S6). We hypothesize that domestication decreased the microbial diversity of the tomato root microbiome compared with that of grasses growing in the same soil. Plant domestication is targeted at meeting the requirements of humans, thereby decreasing plant genetic variability and generating crops dependent on humans (Doebley et al., 2006; Bulgarelli et al., 2013). Interestingly, the two-step model for root microbiota resembles the effects of reductive gene diversity in crop domestication (Doebley et al., 2006). Current agricultural management includes practices such as fertilizer-driven production, which decreases the importance of plant-microbe interactions when scavenging for nutrients (van der Heijden et al., 2008). Although it is not as descriptive with metagenome-predicted proteins, and it probably needs further refinement, maybe through linking the OTU abundance with pan-genomics and metagenomics to describe the genomic coding diversity (Delmont and Eren, 2018). To test the hypothesis that the tomato predicted metaproteome is divergent from those of the soil and ruderal plants, as suggested by the 16S α-diversity dendrogram (Figure 3), we performed a constrained analysis of principal coordinates (CAP) ordination (Figure 4C). We used the protein abundance as CAP input, and we constrained the analysis by sample type, pH, total N, C, and P. This metagenomic profiling of the microbial communities showed that ruderal plants and soils have a similar composition of predicted proteins (Figure 4C), differentiating them from tomato rhizospheres and highlighting the host-dependent selection. The CAP explained 16.6% of the total observed variance, with CAP1 (9.4%) splitting the tomatoes from ruderals and source soils. Ruderal plants were closer to the soils, but not mixed with them (CAP, Bray-Curtis distance, PERMANOVA 9,999 permutations, *p* < 1e-4). The split tomato and ruderal-soil groups are also supported by ANOSIM (*R* = 0.4568; *p* < 0.001; 999 permutations) (Figure 4C). Correlations with the measured geochemical variables with the two CAP axes showed positive correlations of P, source soils (SI), and ruderal plants (RZ), while negative correlations observed for pH, N, and C (Figure 4C).

### Shotgun Metagenomics Taxa Assignments

We were able to bin and classify 38% ± 1.66 of the sequenced metagenomic reads to multiple taxa. Bacteria accounted for 88.14% of the identified matches, followed by 7.09% of Eukaryota, with almost half of the hits corresponding to Fungi (3.83%) (Supplementary Table S10). Further work will explore eukaryotic, archaeal, and viral diversity of the sequenced metagenomes. In soils, the most abundant bacterial species binned was *Solirubrobacter soli* (Actinobacteria), which was also highly abundant in ruderals and tomatoes. *Sphingomonas* sp. URHD0057 (α-Proteobacteria) were most abundant in ruderals, along with *Solirubrobacter soli* and the Rhizobiales *Rhodoplanes* sp. Z2-YC6860. The 16S data showed that Bacteroidetes were significantly enriched in tomato roots compared to soil and ruderals; metagenomic bins confirm the 16S rRNA gene trends. There are reports about bacterial groups’ enrichment, such as Bacteroidetes on wild plants and Proteobacteria on domesticated plants (Pérez-Jaramillo et al., 2018). Within the principal tomato metagenomic bins, we found *Ohtaekwangia koreensis*, *Flavobacterium terrae, Niastella vici, Chryseolinea serpens* the metagenome-assembled genome of a *Chitinophagaceae* bacterium IBVUCB2 as Bacteroidetes species.

**FIGURE 5:**
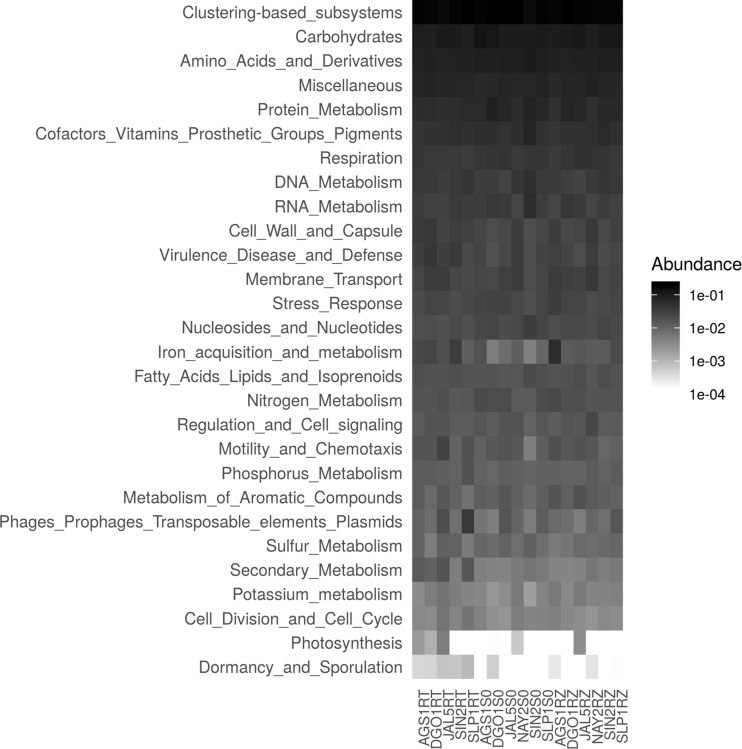
Summary of metagenomic functional profiles. Heatmap is describing the level 1 SEED subsystems ontology annotations in each row. Although only using the 138,627 M5NR matches to the SEED, representing 4.4% of the total dataset, it was helpful to describe the main molecular functions. Columns represent each metagenome, and the first four alphanumeric codes are for location; suffixes indicate sample type: RT is tomato rhizosphere metagenome, SI is source soil, and RZ represents ruderal plant rhizosphere metagenome.

We analyzed a functional summary of the sequenced metagenomes using the SEED subsystem gene ontology (Figure 5). The largest category was clustering-based subsystems, which include protein families that are quite diverse from the CRISPR, sugar metabolism, other known categories, and hypothetical proteins. We only found small differences (Tukey’s HSD) in iron acquisition metabolism (*p* = 0.07), cell wall and capsule genes (*p* = 0.06) between soils and tomatoes. We found significant differences (*p* = 0.017) between ruderals and tomatoes in sulfur metabolism genes (Figure 5).

### Enriched Proteins in the Rhizospheres-Soil Comparison

**FIGURE 6:**
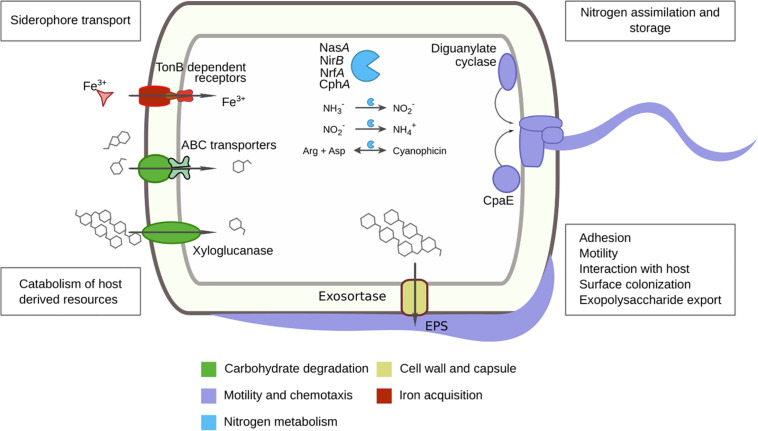
Tomato rhizosphere core metagenome and differential features with soil and ruderal plants summary. Predicted tomato core proteins and enriched tomato proteins are color-coded to the related cellular processes. The found proteins were previously reported as fundamental for plant-microbe interactions.

Pairwise comparisons were made using DESeq2 to find significant (*p* < 0.001, Bonferroni) predicted protein enrichments. Comparing tomatoes (RT) and soils (SI), we identified 67 enriched proteins in RT involved in motility, chemotaxis, and biofilm formation (e.g., LuxR, CheY, diguanylate cyclase, CpaE), complex carbohydrate degradation (e.g., xyloglucanase, cellulase Cel5F), antibiotic resistance (e.g., β-lactamase class C), iron metabolism (e.g., TonB), and sporulation (e.g., SpoIIIE), as well as secretion system-related proteins (e.g., exo-sortase) (Supplementary Figure S7). The enrichment of Proteobacteria in tomato is in line with enriched genes such as motility and chemotaxis, widely distributed amongst α, β, and δ-Proteobacteria (Liu and Ochman, 2007). Motility traits are important for host colonization; this has been tested by mutagenesis in *Pseudomonas fluorescens* WCS36, reducing colonization efficiency of plant roots (de Weert et al., 2002), also reported for *P. fluorescens* SBW25 (Turnbull et al., 2001). Diguanylate cyclase and CpaE are involved in biofilm formation and pili production in *Caulobacter crescentus* (Skerker and Shapiro, 2000; Del Medico et al., 2020). Another interesting metabolic feature relevant for the plant-associated niche found in tomato roots is the enzyme xyloglucanase, involved in the degradation of xyloglucan. This heteropolysaccharide comprises up to one-quarter of the total carbohydrate content of terrestrial plant cell walls (Scheller and Ulvskov, 2010; Figure 6).

When comparing ruderals (RZ) and tomatoes (RT), we found 16 enriched proteins in RT and 11 in RZ (Supplementary Figure S8). The lowest number of enriched proteins between tomatoes and ruderals indicates that their shared set contains common features in plant-microbe interactions. Compared to RT, the RZ-enriched proteins included transporters and, interestingly, osmotic sensor components (e.g., osmosensitive K channel histidine kinase). The RT-enriched proteins, compared to RZ, included several peptidases (e.g., M17 leucyl aminopeptidase) and some horizontal gene transfer elements (e.g., integrase-recombinase, ISRSO17 transposase, bacteriophage N4 adsorption protein B). Interestingly, multiple similar proteins enriched in the RT-SI comparison were also enriched in the RT-RZ (e.g., β class C, glycoside hydrolases), remarking the host genotype filtering of RT. Finally, comparing RZ-SI registered only two RZ-enriched proteins, indicating the similarities between soil and ruderals (Supplementary Figure S9). The full list of overrepresented proteins for each comparison is available (Supplementary Table S11).

### The Tomato Rhizosphere, Soil, and Ruderal Plant Core Metagenomes

It seems that the tomato was highly selective about its microbial inhabitants; we found 2,762 protein families ubiquitous in all tomato roots tested (Supplementary Figure S10). We used the protein annotation to reduce the dataset to 1,777 core proteins and only 1,353 exclusively in tomato (Supplementary Table S12). The core tomato metagenome was contrasting to the soil with only 162/639 and the ruderal metagenome with just 143/694 core-exclusive proteins. Some essential proteins were expected to be part of the core metagenomes and worked as controls for our searches, such as ribosomal proteins, DNA and RNA polymerases, gyrases, chaperonin GroEL, and we found them all within the tomato core metagenome. Within the tomato core metagenome, we found multiple strategies to cope with nitrogen, such as regulation genes via denitrification (*nosZ*) and nitrate reductase genes (*nasA, nirB, and nrfA*) to obtain ammonia (Figure 6). The high abundance of Actinobacteria in the source soils and the switch to a Proteobacteria dominance in the FS suggests processes such as biological nitrogen fixation and microbial biomass increments. Both Actinobacteria and Proteobacteria are capable of nitrogen fixation since their genomes contain nitrogenases (Boyd and Peters, 2013). Glutamate, glutamine synthetases, and their transferases were also detected in the RT core metagenome and could regulate amino acid synthesis and ammonia. Additional nitrogen storage proteins were detected, such as cyanophycin synthetase (CphA) and cyanophycinase, within the RT metagenomic core; cyanophycin is a non-ribosomal peptide built by aspartic acid and arginine. This reserve polymer regulates N and C and mediates N storage, providing bacterial fitness advantages under nitrogen fluctuations (Watzer and Forchhammer, 2018; Figure 6). Further, we found allantoinase and allantoate amidohydrolase genes, which are responsible for allantoin degradation to ammonia (Cruz-Ramos et al., 1997; Ma et al., 2016). Patatin-like phospholipase proteins were also found in the tomato core metagenome; they are phospholipases originally described in potato, but with abundant homologs in bacteria (Banerji and Flieger, 2004). Bacteria use patatins to target host cell membrane as effectors via the type III secretion system (Finck-Barbançon et al., 1997; Phillips et al., 2003; Sato et al., 2003) and are activated by ubiquitin (Anderson et al., 2015). The eukaryotic patatins are known to have antimicrobial activities (e.g., *Phytophthora infestans* inhibition) (Bártová et al., 2019). Tomato and potato, belonging to the family *Solanaceae*, interact with microbes via patatin and patatin-like proteins, and we will further explore plant-microbe interactions mediated by these proteins. While significantly enriched in ruderals, leucyl aminopeptidase was also ubiquitous in tomato metagenomes. Interestingly, leucine aminopeptidase A (LapA) is expressed in tomato after wounding and prevents foraging (e.g., *Manduca sexta* foraging tomato) (Fowler et al., 2009). LapA is also transcriptional and protein-responsive to microbial pathogens (Pautot et al., 1993; Pautot et al., 2001). The bacterial leucine aminopeptidases found in tomato metagenomes could be expanding the plant’s defensive response through LapA, but this is yet to be explored. The complete M5NR identifiers and core metagenomes are available (Supplementary Table S13).

Describing the tomato core microbiome and metagenome under multiple soils also allowed us to test the plant genotype filtering effect, evaluating selected microbes in diverse environments. With the current advances in synthetic biology, the tomato core metagenome could lead to a tomato root metagenomic chassis. This core metagenome could lead to microbe-complemented plant breeding programs aiming to reduce and optimize fertilizer use while increasing plant resilience, such as that observed in ruderal plants. Further possibilities could be the recovery of the domesticated missed root microbes from wild plants.

By using 16 geochemically diverse soils as microbial inputs for root colonization, we discarded the role of soil as the major structuring factor of root microbial communities, particularly of their coding genes. Further work is needed for detecting other environmental microbe sources than the soil for rhizosphere metagenomic diversity. Weather-dependent ruderal plant roots are a nutrient and moisture oasis for soil microbial communities with a higher taxonomic α-diversity. The tomato root microbiome followed the two-step model of microbiome acquisition. The reduced total protein number, along with significant enrichments in the tomato root metagenomes compared to ruderals and soils, suggests a tomato rhizosphere specialization and a possible domestication trade-off. Plants had been domesticated since the Neolithic age some 10,000 years ago (Purugganan and Fuller, 2009), and genomic changes in microbes linked to domestication processes have been documented (genome reduction, insertion sequences, and transposition expansions), such as the enriched genes found in RT (Mira et al., 2006). Our experimental setup showed that tomato enriched plant-microbe interaction genes (Figure 6). Altogether, our results show that tomato roots have a convergent, genotype driven, and reduced microbiome compared to their source soils, following the two-step selection model for the root microbiome. This is contrary to the ruderal plants, which exhibit a larger microbiome diversity than their soils, not following the two-step model.

## Materials and Methods

### Soil and Local Plant Roots Sampling

Edaphological charts were used to locate eight different soil groups, according to the United Nations FAO classification (IUSS Working Group WRB, 2015) from 16 different geographic locations described in Figure 1A and Table 1. In each location, 0.09 m^1^ quadrats were placed, and duplicate root samples were taken from the quadrant dominating plant species and the soil below them. We collected 2 kg of each soil into sterile plastic bags for the common garden experiment and biogeochemical analysis. All soil samples were taken from a depth not larger than 10 cm. The soil was kept at 4°C in a darkroom until greenhouse experiments were conducted 1 month later. *In situ* soils were collected for each soil group, poured into duplicate sterile centrifuge tubes (50 mL volume), and immediately field frozen in liquid nitrogen until storage into a −80°C freezer metagenomic DNA extraction.

### Common Garden Experiment, Harvesting, and Sample Collection

The tomato seeds used were *Solanum lycopersicum* L. Cv. *Río grande* (Sun Seeds, Parma, ID, United States). Seeds were surface disinfected in 70% ethanol for 1 min, followed by a wash in 2.5% NaOCl for 2 min, and rinsed with sterile distilled water. Seeds were germinated in 1% agar for 96 h in a dark growth chamber at 27°C. Sprouts were aseptically transplanted into duplicated pots filled with the collected soils, two plants per pot were transplanted, summing four biological replicates; additionally, pots with each soil were prepared without plants which served as a control to track the changes in the soil microbiome composition due to the treatment applied in the greenhouse lacking the influence of plant development (US; Figure 1C). Pots were set in the greenhouse randomly, and plants were watered with tap water every other day and harvested after 60 days of growth. All soil samples (Figure 1) were collected in 50 mL sterile tubes and frozen at −80°C until metagenomic DNA extraction. Roots were separated from shoots to collect rhizosphere and endosphere samples by removing loose soil, followed by a washing and ultrasound procedure in 1X PBS buffer (137 mM NaCl; 2.7 mM KCl; 10 mM Na_2_HPO_4_; 1.8 mM KH_2_PO_4_) as described before (Lundberg et al., 2012). Tomato rhizosphere and endosphere metagenomic pellets were recovered through centrifugation (50mL tubes centrifuged at 1,300 g during10 min). Roots and shoots were oven-dried at 60°C for 24 h to measure plant biomass production. Due to low DNA extraction efficiency by this method in ruderal plant roots, they were cut and separated into ten 1.5 mL tubes, which received the same treatment as the 50 mL tubes. All sample pellets were frozen and kept −80°C until metagenomic DNA extraction.

### Soil Geochemical Analyses

Initial and final soils were oven-dried for 24 h at 70°C. The pH was measured in deionized water (1:4 w:v) with a Corning digital pH meter. Total carbon was measured by coulometric combustion detection (Huffman, 1977) with a Total Carbon Analyzer (UIC Mod. CM 5012; Chicago, IL, United States). Total nitrogen was determined by a semi-Kjeldahl method and phosphorus by the molybdate colorimetric method after ascorbic acid reduction (Murphy and Riley, 1962) using a Bran-Luebbe Auto Analyzer III (Norderstedt, Germany). The Lang’s aridity index (Lang, 1920) of each site was calculated using historical data of mean annual precipitation and temperature for each sampling location, and data was consulted at the Atmospheric Sciences Center^2^ of UNAM. Non-metric multidimensional scaling (NMDS) of the samples was calculated with the geochemical data using the metaMDS function in the vegan R package (Oksanen et al., 2015) and plotted with ggplot2 (Wickham, 2009). Detailed statistical and bioinformatic methods are available at Github^2^.

### Metagenomic DNA Processing and Massive Sequencing

The metagenomic DNA of all samples was extracted using the Mobio PowerSoil DNA extraction kit (MoBio, Carlsbad, CA, United States), following the manufacturer’s instructions. Briefly, for soils, ∼0.25 g were used for the extraction, for rhizosphere and endosphere pellets collected after washing and sonication of the roots were used respectively, as previously described (Lundberg et al., 2012). Then, the Mobio protocol was slightly modified to get extra DNA by heating the C6 elution solution to 60°C before eluting the DNA, and two 30 μL elutions were performed on the same spin filter. The same DNA was used for both amplicon and whole metagenome shotgun sequencing.

PCR amplification of the 16S rRNA gene was performed in duplicates, followed by the Illumina^®^ MiSeq protocol for 16S metagenomic sequencing library preparation (Illumina 2013) using the 341F/805R primer pair targeting the V3-V4 regions with the Illumina sequencing adaptors in 5′ (341F: 5′-CCTACGGGNGGCWGCAG*-*3′; 805R: 5′-ACTACHVGGGTATCTAATCC 3′). PCR reactions were performed in a 20 μL volume, consisting of 0.16 μL *Pfx* polymerase (0.02U/μL) (Invitrogen, Thermo Fisher Scientific, Waltham, MA) 2μL buffer, 3 μL enhancer, 1.2 μL of each primer (5μM), 1.6 μL dNTPs (2.5 mM), 0.6 μL Mg2S04 (1.5μM), 9.2 μL PCR grade water and 2 μL DNA template. The PCR program for amplification was 95°C for 3 min, followed by five cycles of 94°C for 30 s, 55°C for 30 s, 68°C for the 30 s, followed by 25 cycles of 94°C for 5 s and 68°C for 30 s. The duplicate amplification products of each sample were pooled and purified with the SV Wizard PCR Purification kit (Promega, Madison, WI) following the manufacturer’s instructions. Amplicon library sequencing was done in the Illumina^®^ MiSeq platform in a 2 × 300 paired-end configuration at the University Unit of Massive Sequencing and Bioinformatics^3^ of the Biotechnology Institute, UNAM, Mexico. Whole shotgun metagenome sequencing libraries were prepared using the Truseq PCR free library preparation kit for selected initial soils, ruderal plants, and *S. lycopersicum* rhizospheres, which were then sequenced with an Illumina HiSeq 2000 in a 2 × 100 bp reads, at the facilities of Macrogen, Korea^4^.

### 16S rRNA Gene Amplicon Sequence Analysis

The 16S rRNA protocol used in this work had been used previously and is detailed at GitHub^5^ (Alcaraz et al., 2018). In summary, gene amplicon libraries were quality inspected using Fastx Toolkit^6^ and trimmed to a 250 bp length. Trimmed paired-end reads were assembled using Pandaseq (Masella et al., 2012). The assembly was performed using a minimum overlap of 15 bp, the minimum output length of 250 bp, the maximum output length of 470 bp, and an alignment threshold of 95%. Finally, assembled sequences were filtered using a minimum PHRED score of 20. All the samples were concatenated and clustered into OTUs, using a 97% identity threshold with *cd-hit-est* (Li and Godzik, 2006). The taxonomy of representative sequences was assigned against Greengenes (De Santis et al., 2006) database with QIIME’s scripts (Caporaso et al., 2010). After taxonomic classification, singletons, and chimeras were removed as well as sequences corresponding to the mitochondria, chloroplast, and unassigned hits were filtered out. Finally, the representative OTU sequences were aligned with SSU-align (Nawrocki et al., 2009), and a phylogenetic tree was constructed with Fasttree (Price et al., 2009). Detailed statistical and bioinformatic methods are available at Github (See text footnote 2).

### Metagenomic Shotgun Sequence Analysis

The quality control of whole shotgun metagenome sequences was done using Trimmomatic (Bolger et al., 2014), only paired-end matched reads were used for subsequent analysis. We filtered out metagenomic reads matching *S. lycopersicum* genome (NCBI BioProject: PRJNA66163), while soils and ruderal plants rhizosphere libraries were filtered against the *Oryza sativa* genome (NCBI BioProject: PRJNA122) with Bowtie2 (Langmead and Salzberg, 2012). Quality and host filtered metagenomic libraries were used to assemble individual metagenomes with metaSPADES (Nurk et al., 2017). High-quality reads were mapped against the metaSPADES contigs, and unmapped reads were subjected to a second assembly with Velvet (Zerbino and Birney, 2008). The resulting contigs from both assemblies were merged and used to predict ORFs and coding proteins with Prodigal (Hyatt et al., 2010). Annotation of predicted proteins was made against the M5NR database (Wilke et al., 2012) using DIAMOND (Buchfink et al., 2015) with the following parameters -f6 -e 1e-10 -k 10 -p1, retrieving Refseq (Pruitt et al., 2007) and SEED subsystems (Overbeek et al., 2014) annotations from M5NR matched identifiers. The abundance of each predicted protein was calculated by mapping the high-quality reads against the predicted ORFs with Bowtie2. All the predicted proteins were clustered using cd-hit (Li and Godzik, 2006) using a 70% identity threshold, and they were parsed into a biom formatted matrix, used as input for sets comparison using UpSetR (Conway et al., 2017). The binning of whole shotgun metagenomic reads was performed with Kaiju (Menzel et al., 2016). Detailed statistical and bioinformatic methods are available at Github^2^.

### Diversity Analysis

The α and β-diversity of soils, rhizospheres, and endospheres from each site were calculated with phyloseq (McMurdie and Holmes, 2013), and vegan R (Oksanen et al., 2015) packages. Taxonomic α-diversity was assessed using a weighted Unifrac (Lozupone et al., 2006) distance matrix. Microbiomes were then hierarchically clustered with the *hclust* method using complete distances and clustering evaluated through the ANOSIM function. OTUs were clustered at the genus level, and Venn diagrams were used to compare the complete root system (rhizosphere + endosphere) microbiome composition of ruderal plants, *S. lycopersicum*, and initial soils using a web Venn diagram calculator^7^. Unique soil, ruderal plants, *S. lycopersicum*, and the ruderal plants-*S. lycopersicum* intersection taxonomic profiles were described at the phylum level based on OTU abundances.

Metabolic α-diversity was estimated through a constrained analysis of principal coordinates (CAP) analysis using Bray-Curtis dissimilarity based on the total abundance of predicted proteins. Differential OTUs and protein abundances comparing rhizospheres or endosphere against soils were calculated using DESeq2 (Love et al., 2014) with a Wald statistical test and a local fit of the data. For 16S rRNA data, OTUs were considered differentially abundant between groups using a *p* < 0.01, for metagenome predicted proteins, a *p* < 0.001 was used as a cut-off. Their 16S rRNA matches identified the collected ruderal plant species to NCBI’s NR database representing a variety of 5 different plant families, mainly grasses (Poaceae *N* = 10, Asteraceae *N* = 3, Lamiaceae *N* = 1, Fabaceae *N* = 1, and Fagales *N* = 1; Supplementary Figure S1). Detailed statistical and bioinformatic methods are available at Github^2^.

## Data Availability Statement

The datasets generated for this study can be found in the NCBI SRA: https://www.ncbi.nlm.nih.gov/sra/PRJNA603590, https://www.ncbi.nlm.nih.gov/sra/PRJNA603586, https://www.ncbi.nlm.nih.gov/sra/PRJNA603603.

## Author Contributions

HB, LS-G, MP, RC-O, FG-O, and LA conceived and designed the research. HB and LA wrote the manuscript. HB, SM-S, MP, and LA performed the field sampling. HB and SM-S performed the experiments. HB, SM-S, CH, MR, and LA analyzed the data. MP, RC-O, FG-O, and LA provided the reagents and material. HB, MP, and LA created the figures. All the authors read and approved the manuscript.

## Conflict of Interest

The authors declare that the research was conducted in the absence of any commercial or financial relationships that could be construed as a potential conflict of interest.

## Footnotes

1 https://github.com/genomica-fciencias-unam/Barajas-2020

2 http://uniatmos.atmosfera.unam.mx/ACDM/

3 http://www.uusmd.unam.mx

4 https://www.macrogen.com

5 https://genomica-fciencias-unam.github.io/SOP/

6 http://hannonlab.cshl.edu/fastx_toolkit/

7 https://bioinformatics.psb.ugent.be/webtools/Venn/

## Supplementary Material

Complete Supplementary Material available at: https://github.com/genomica-fciencias-unam/Barajas-2020.

## Abbreviations

EC: ruderal plant endosphere
ECT: tomato endosphere
FS: final soil
RT: tomato rhizosphere
RZ: ruderal plant rhizosphere
SI: Initial source soil
US: control unplanted soil.
