# Supplementary material for: Testing the Two-Step Model of Plant Root Microbiome Acquisition Under Multiple Plant Species and Soil Sources
Source: Front Microbiol. 2020 Oct 9;11:542742. doi: 10.3389/fmicb.2020.542742 (PMC7581803; doi:10.3389/fmicb.2020.542742)
Supplement: Supplementary file 1 [file Data_Sheet_1.PDF]

Supplementary Material

1 Supplementary Figures

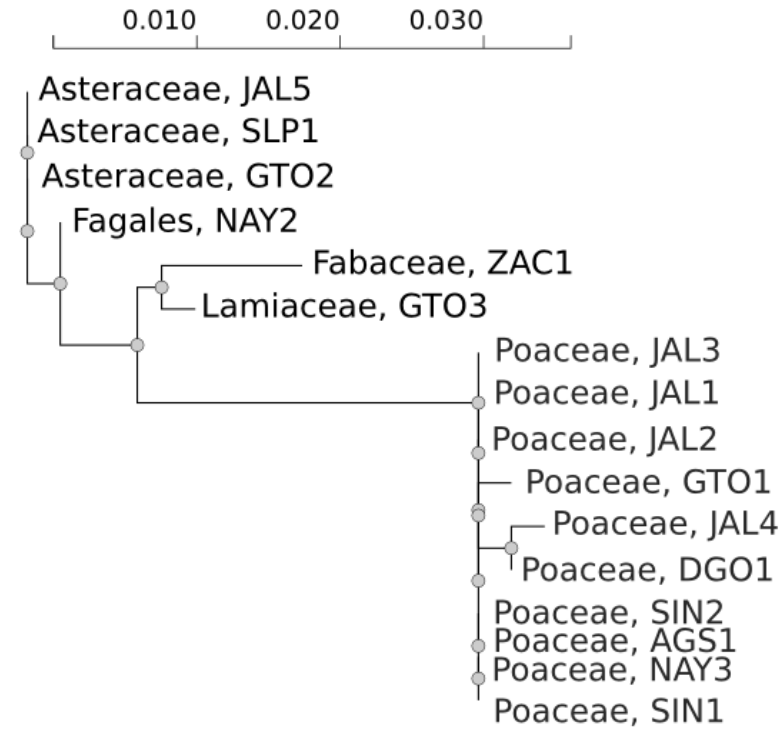

Figure S1. Taxonomic identification of the ruderal plants using the 16S rRNA gene. The maximum likelihood phylogeny and best hit classification showed that the ruderal plants collected in this work, were mainly grasses (*Poaceae*, N=11), composite (*Asteraceae*, N=3), and then single representatives of *Fagales*, *Fabaceae*, and *Lamiaceae*.

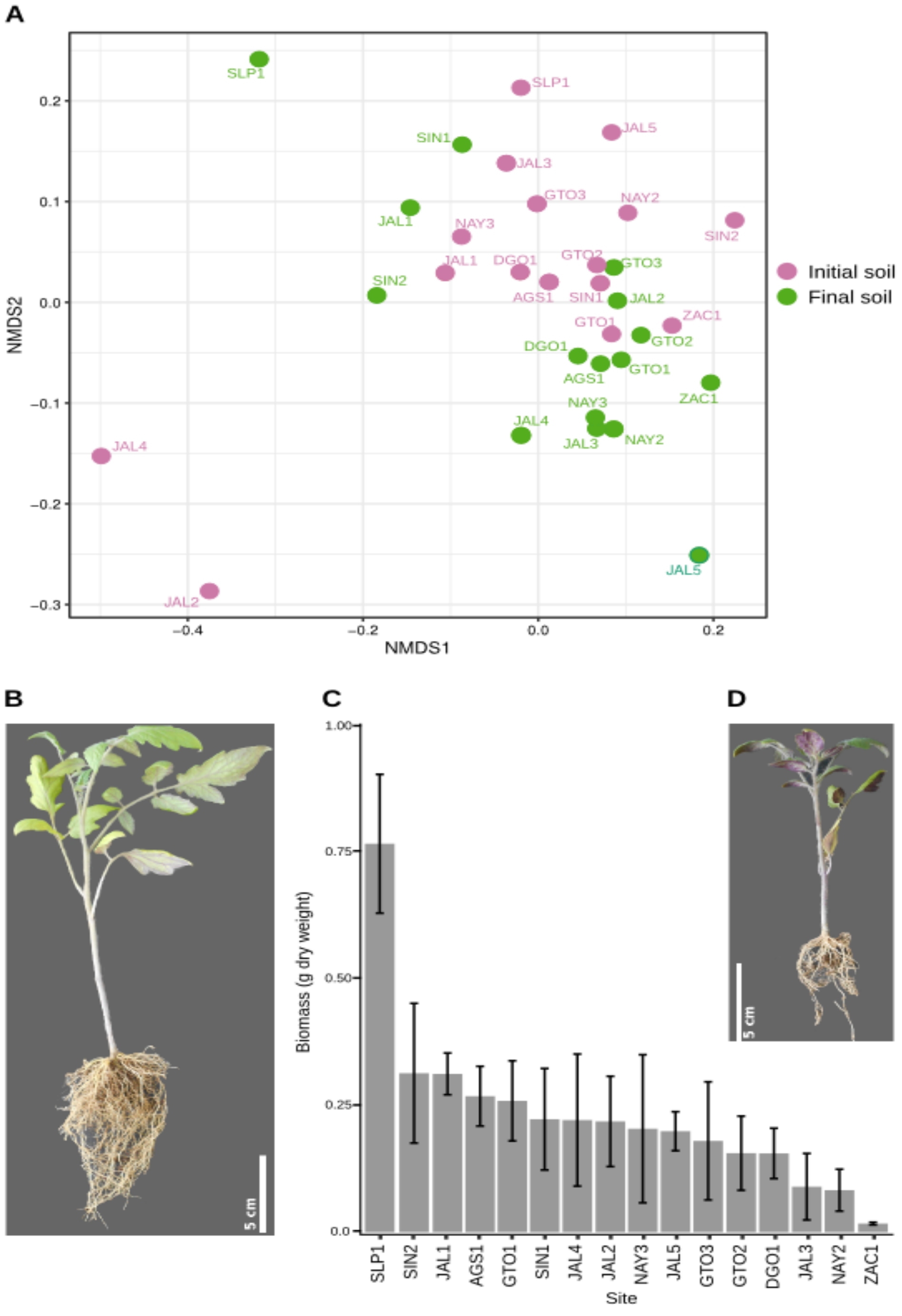

Figure S2. Geochemical diversity of source and final soil, and plant biomass production in common garden experiment. A) NMDS ordination bi-plot of initial soils (SI) and final soils (FS) calculated with the following soil abiotic properties: Aridity index, total carbon content, total nitrogen content, total phosphorus content. NMDS stress=0.116. **B)** Example of tomato individual grown on SLP1 soil. **C)** Bar-plot showing average biomass production of tomato plants in the common garden experiment. **D)** Example of tomato individual grown on NAY2 soil.

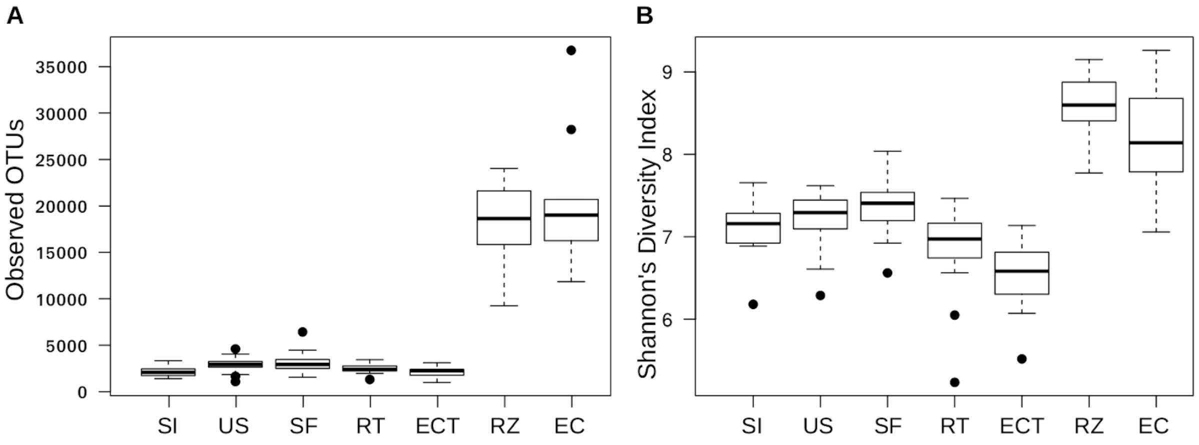

Figure S3. Richness and diversity of initial, final, and unplanted soil, tomato, and ruderal plants rhizosphere and endosphere. Boxplots showing median values of **A)** Observed OTUs and **B)** Shannon diversity index. SI=initial soil, FS=Final soil, US=unplanted soil, RT=tomato rhizosphere, ECT=tomato endosphere, RZ=ruderal plants rhizosphere, EC=ruderal plants endosphere.

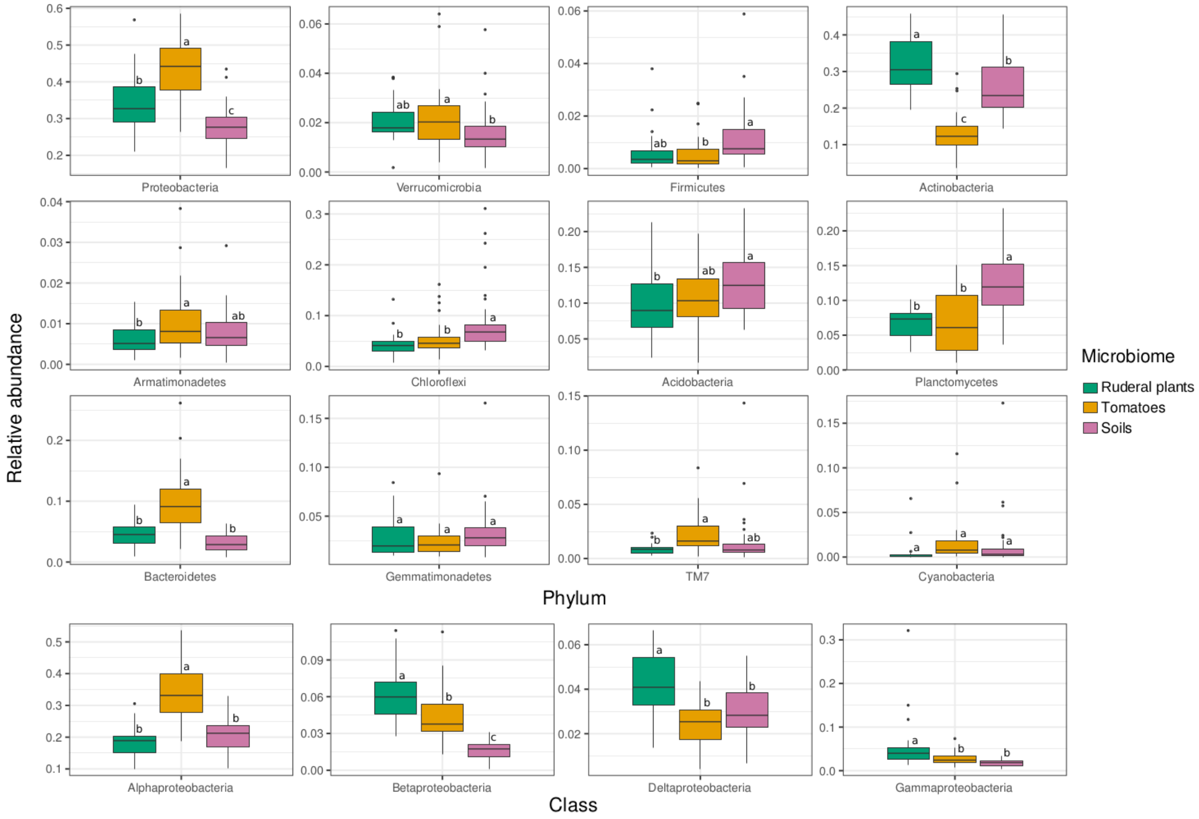

Figure S4. Relative abundance of bacterial phyla in soils, ruderal plants and tomato roots. Each panel shows the relative abundance of different phyla. Proteobacteria are shown at the class level.

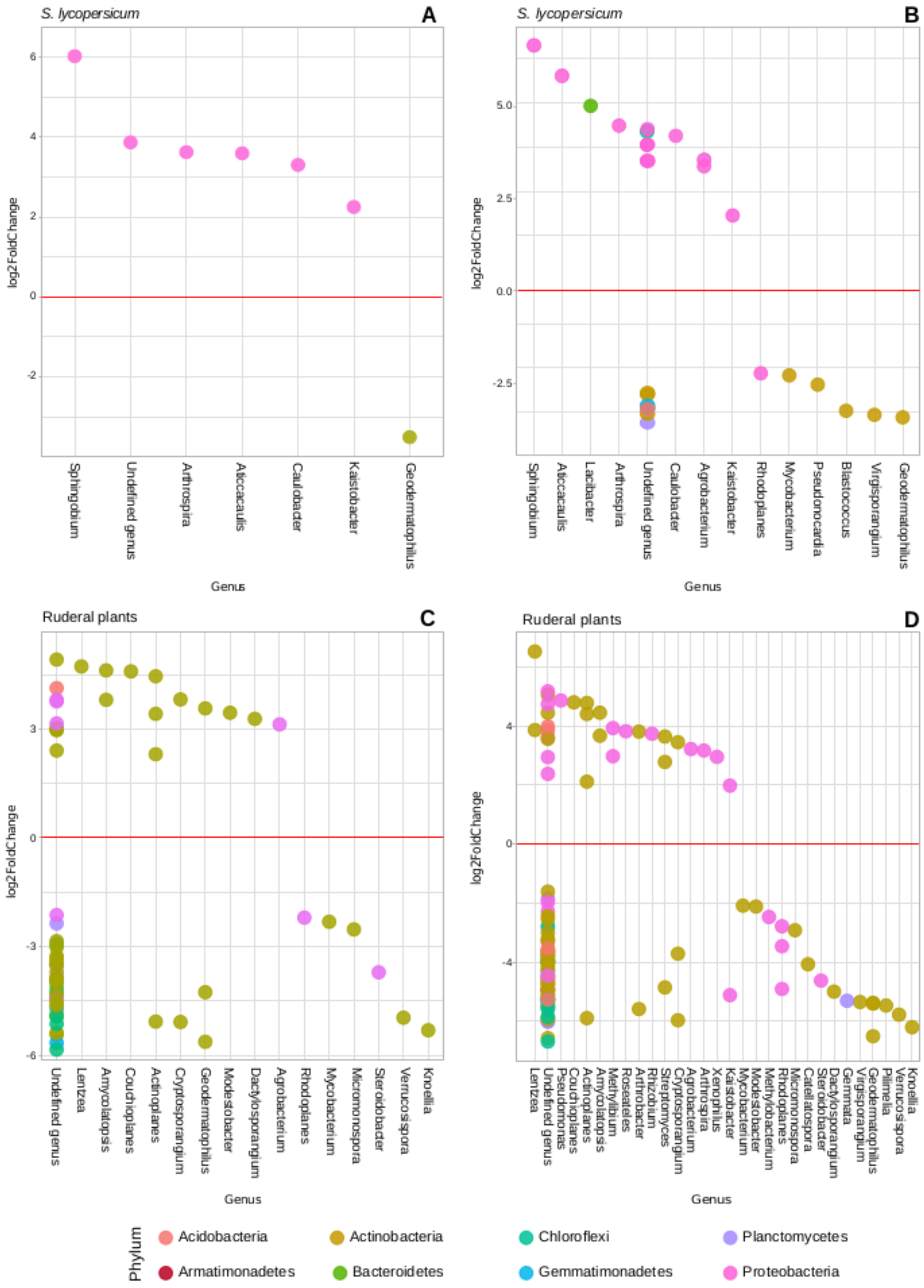

Figure S5. Enriched OTUs in tomato and ruderal plants root systems. Log2 Fold Change of OTUS abundance in comparisons between initial soils and **A)** Tomato rhizospheres. **B)** Tomato endosphere. **C)** Ruderal plants rhizosphere. **D)** Ruderal plants endosphere. Positive Log2 Fold Change values indicate differentially abundant OTUs in plant root systems.

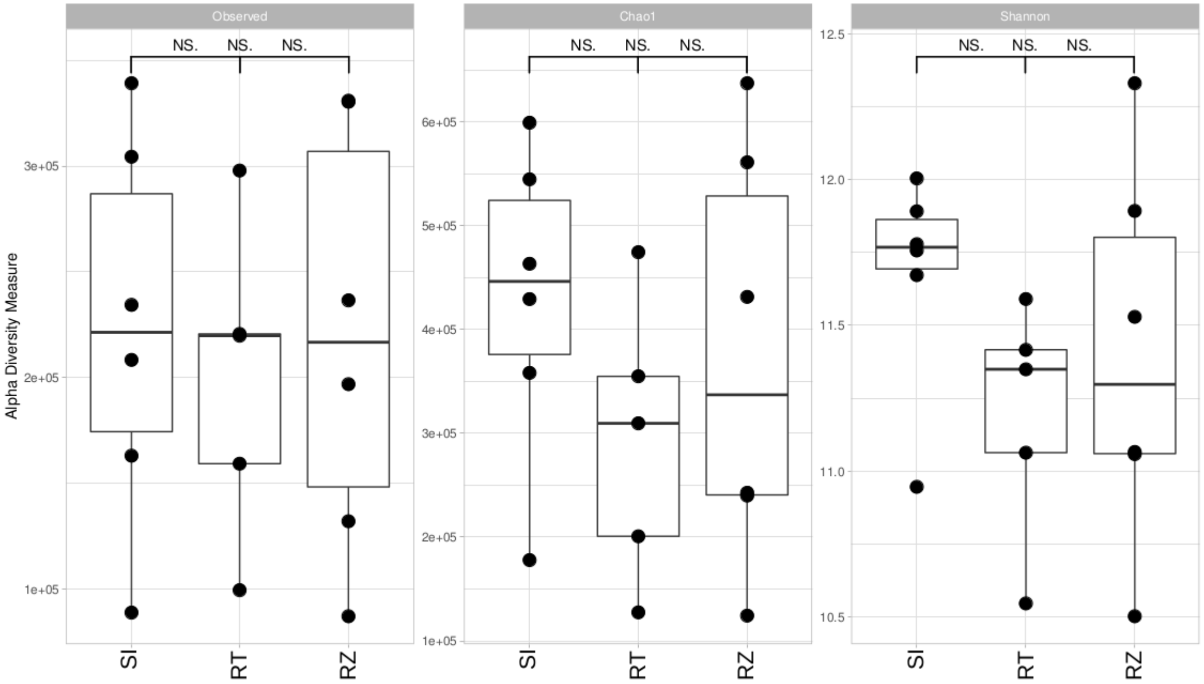

Figure S6. Alpha diversity of predicted proteins in soil sources, tomato, and ruderal plants metagenomes. The number of observed proteins, Chao1, and Shannon diversity index are shown. No significant differences were found between groups.

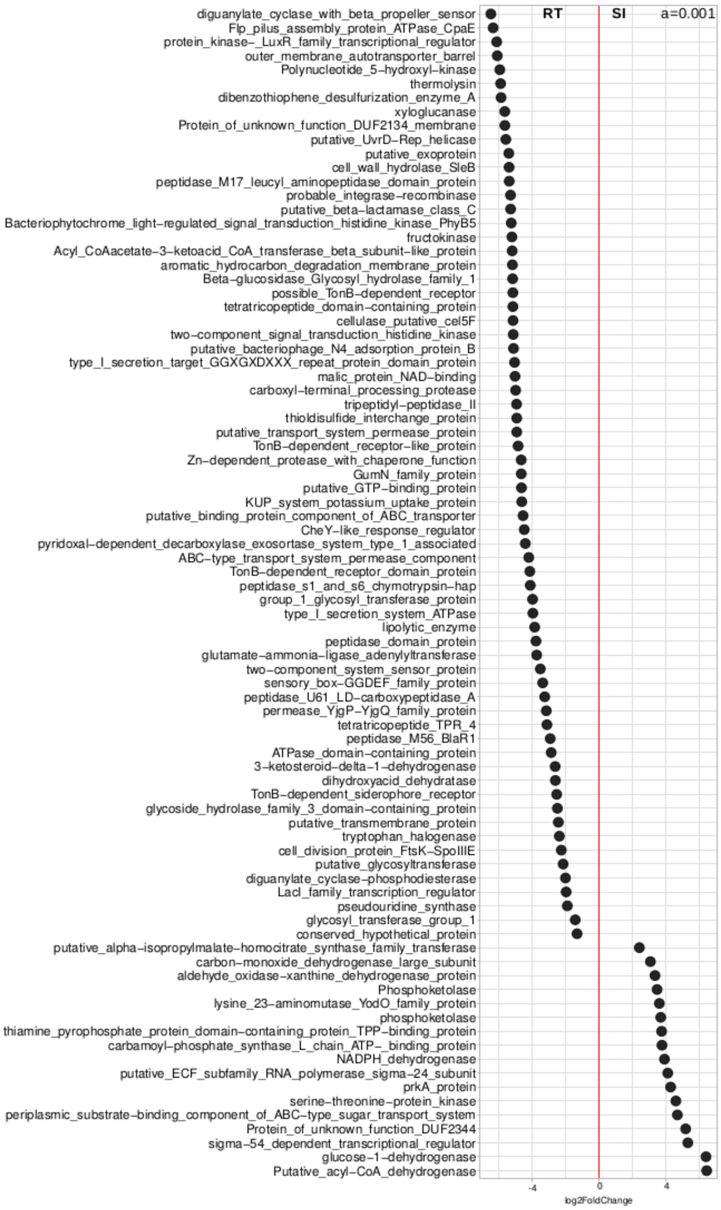

Figure S7. Differentially abundant proteins in tomato rhizosphere against soil metagenomes. Log2 fold change values ($p < 0.001$) of annotated proteins are shown. Sixty-four proteins were differentially abundant in tomato rhizosphere. Negative log2 fold change values are tomato rhizosphere enriched proteins, while positive values represent soil enriched proteins.

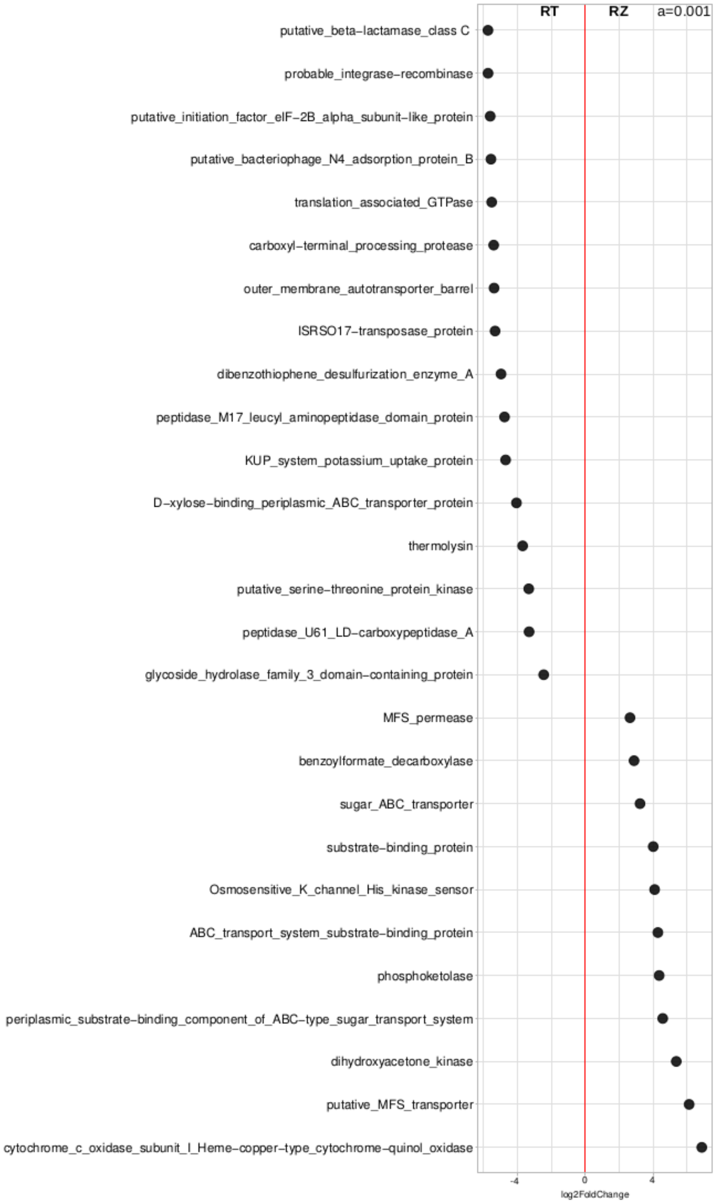

Figure S8. Differentially abundant proteins in the comparison between ruderal plants and tomato rhizosphere metagenomes. Log2 fold change ($p < 0.001$) of annotated proteins are shown. Sixteen proteins were differentially abundant in tomatoes and eleven in ruderal plants rhizospheres. Negative log2 fold change values are tomato rhizosphere enriched proteins, while positive values are enrichments in ruderal plants.

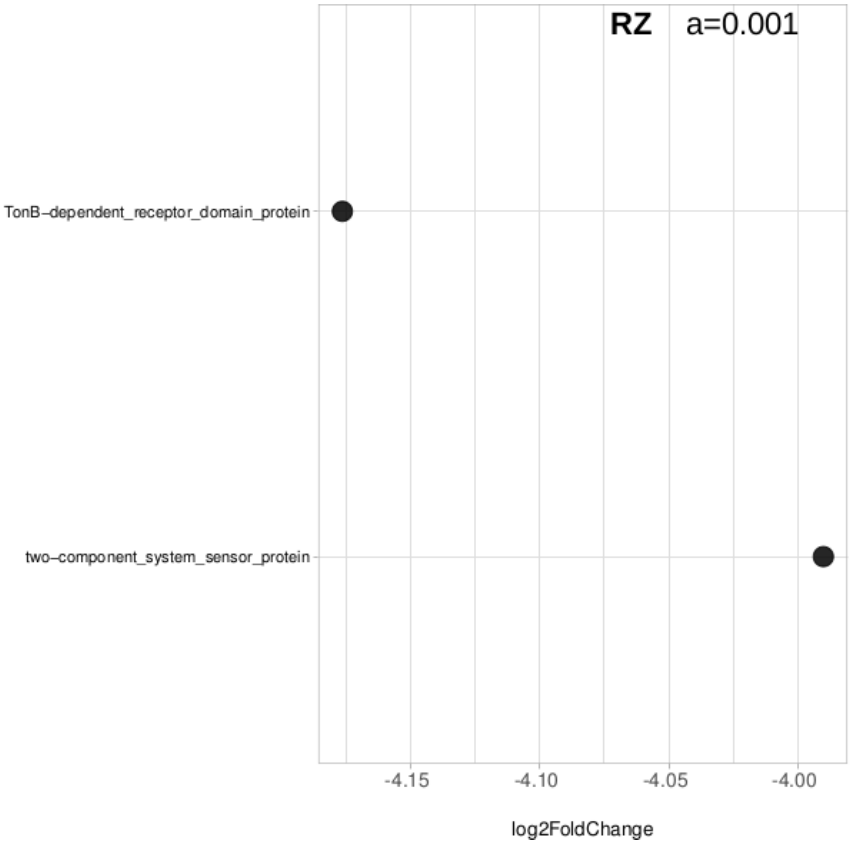

Figure S9. Differentially abundant proteins in ruderal plants rhizosphere against initial soil metagenomes. Log2 fold change ($p < 0.001$) of annotated proteins are shown. Only two proteins were differentially abundant in ruderal plants' rhizosphere. Negative Log2 fold change values are proteins enriched in ruderal plants, positive are enriched in soil.

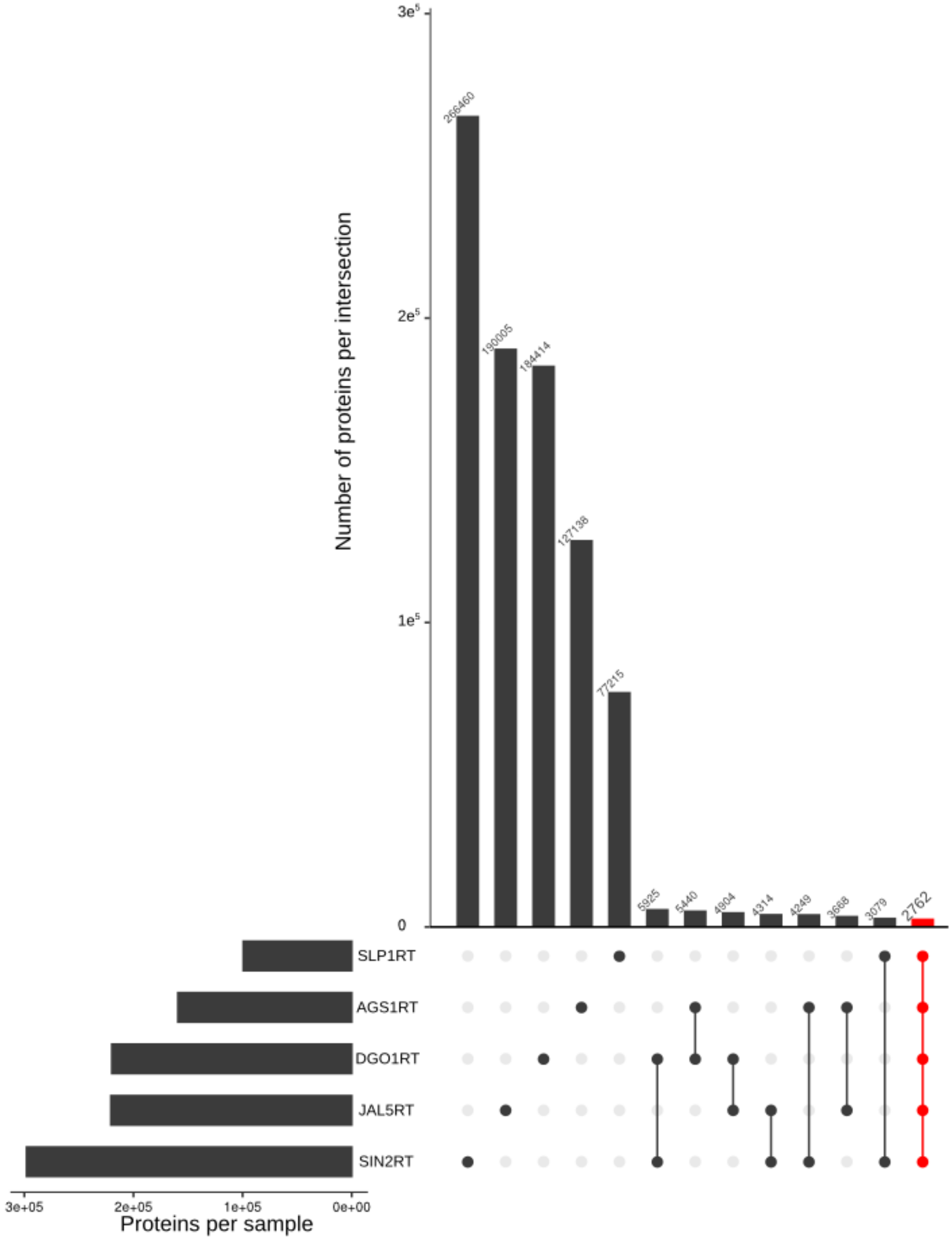

Figure S10. Tomato core metagenome. Upset diagram showing unique and shared sets of proteins in the tomato rhizosphere metagenomes. The tomato core metagenome consists of 2,762 proteins.

2 Supplementary tables

Complete supplemental material available at:

https://github.com/genomica-fciencias-unam/Barajas-2020

Supplementary Table S1. Summary of paired-end reads and assembled sequences in 16S rRNA gene libraries.

Supplementary Table S2. Diversity metrics of microbiome samples.

Supplementary Table S3. Unique and shared OTUs between tomato, ruderal plants and soils.

Supplementary Table S4. Pairwise cophenetic distances between microbiomes.

Supplementary Table S5. Phylum relative abundance in soils, rhizospheres and endospheres of tomato and ruderal plants.

Supplementary Table S6. Deseq2 enriched OTUs in soils, rhizospheres and endospheres of tomato and ruderal plants.

Supplementary Table S7. Summary of whole shotgun metagenomes sequencing and assembly.

Supplementary Table S8. Shared and unique predicted proteins between tomato, ruderal plants and soils metagenomes.

Supplementary Table S9. Whole shotgun metagenomes α-diversity metrics.

Supplementary Table S10. Abundance of taxa based on binning of metagenomic reads with Kaiju.

Supplementary Table S11. Deseq2 enriched proteins in soils, rhizospheres and endospheres of tomato and ruderal plants.

Supplementary Table S12. Soils, tomato, and ruderal plants rhizospheres core metagenomes.

Supplementary Table S13. Shared and unique proteins between soil, tomato, and ruderal plants core metagenomes**.**
